# Unique evidence of fluid alteration in the Kakowa (L6) ordinary chondrite

**DOI:** 10.1038/s41598-022-09465-6

**Published:** 2022-04-12

**Authors:** I. P. Baziotis, C. Ma, Y. Guan, L. Ferrière, S. Xydous, J. Hu, M. A. Kipp, F. L. H. Tissot, P. D. Asimow

**Affiliations:** 1 Agricultural University of Athens, Iera Odos 75, 11755 Athens, Greece; 2 Division of Geological and Planetary Sciences, California Institute of Technology, Pasadena, CA 91125 USA; 3 Natural History Museum Vienna, Burgring 7, 1010 Vienna, Austria; 4 The Isotoparium, Division of Geological and Planetary Sciences, California Institute of Technology, Pasadena, CA 91125 USA

**Keywords:** Meteoritics, Mineralogy

## Abstract

Meteorites preserve evidence of processes on their parent bodies, including alteration, metamorphism, and shock events. Here we show that the Kakowa (L6) ordinary chondrite (OC) preserves both shock-melt veins and pockets of detrital grains from a brecciated and altered object, including corundum, albite, silica, fayalite, forsterite, and margarite in a Pb- and Fe-rich matrix. Preservation of the observed mineralogy and texture requires a sequence of at least two impacts: first, a high-velocity collision formed the shock melt veins containing the high-pressure minerals ringwoodite, wadsleyite, majorite, and albitic jadeite; later, a low-velocity impact formed fractures and filled them with the detrital material. Oxygen and Pb isotope ratios suggest an OC origin for these detrital minerals. Although fluid alteration is common in carbonaceous chondrites, the discovery of margarite with an OC oxygen isotopic signature is novel. Kakowa extends both the impact and alteration history of L6 ordinary chondrites in general.

## Introduction

Meteorites preserve evidence of the modifications that primitive solar system material experienced due to processes such as thermal metamorphism, fluid alteration, and shock damage on their parent bodies. The most direct evidence for the action of liquid water is the preservation of secondary hydrous minerals, which have so far mostly been documented in carbonaceous chondrites^1^. In particular, the oxidized subgroup of CV carbonaceous chondrites is known to contain margarite, vesuvianite, and kaolinite^1,2^. In ordinary chondrites (OCs), the only hydrous secondary phase noted by Brearley^3^ is fine-grained Fe-rich smectite in the unequilibrated meteorites Semarkona (LL3.00) and Bishunpur (LL3.15). The unequilibrated chondrite Tieschitz (H/L3.6) hosts a sodic-calcic amphibole indicating fluid metasomatism at or close to the peak of thermal metamorphism^4^. In more equilibrated OCs, phyllosilicates are even more rare or totally absent, however phases other than phyllosilicates do indicate alteration in these objects. Metasomatic processes are recorded in OCs from types 3.6 to 3.9 by the presence of sodalite, scapolite, and nepheline; and from types 4.0 to 6.0 by albite and K-bearing feldspar^5^.

Many OCs preserve records of impact events due to collision(s) among their parent asteroids^6–10^. Such meteoritic impact records help to constrain the shock conditions and hence parameters of impact events such as encounter velocity and the sizes of impactors and targets. In turn, the co-evolution of planetesimal sizes and their orbital excitation can distinguish among scenarios for the early evolution of the solar system^11^. Shock parameters can be inferred from several lines of evidence, including brecciation, deformation in minerals, and the presence and textural features of melt veins (MVs) that often contain high-pressure (HP) minerals^12–20^. A notable group of meteorites known as polymict breccias contain fragments of multiple objects, presumably derived from both impactor and target of one or more collisions and reassembled as rubble piles^21^. Although such breccias are not unusual, they typically represent low-velocity collisions; polymict breccias from impacts fast enough to form HP minerals are uncommon^22,23^. Although collisions were most common in the early evolution of the solar system, there is strong evidence that the L chondrite parent body was disrupted by a major collision at 470 Ma^24,25^, resulting in debris that continues to dominate the current flux of meteorites to the Earth^26^.

**Figure 1:**
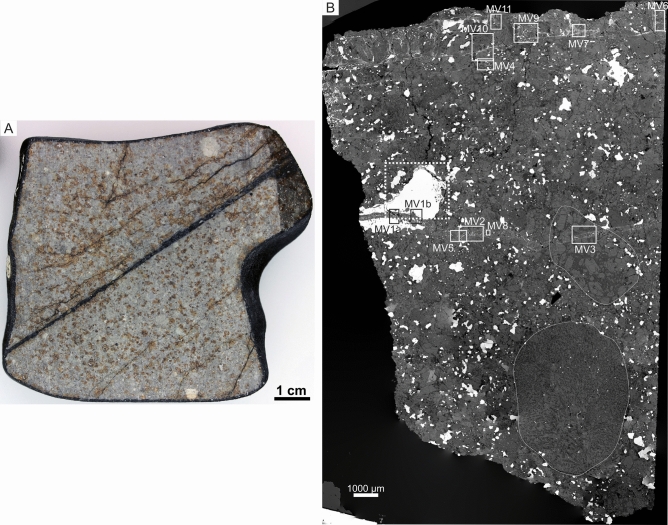
(**A**) Macrophotograph of Kakowa meteorite (NHMV-A557) with a straight melt vein cross-cutting the groundmass. (**B**) Back-scattered electron (BSE) image mosaic of Kakowa section (NHMV-N6231) showing the studied areas within the different melt veins (MVs). A dashed white rectangle shows the area that contains the pockets of exogenous material. Notable are the two large chondrules (delineated by fine white lines), one of them clearly cross-cut by MV3.

Here, we report new data on the historical fall Kakowa, an L6 ordinary chondrite that fell in Romania on May 19th 1858 and was collected within minutes while, according to historical records, still hot^27^. Kakowa is considered to be of shock stage S4–S5 (Fig. 1). We studied its texture, mineralogy, and mineral composition by optical and electron microscopy, electron probe microanalysis (EPMA), micro-Raman spectroscopy, and electron back-scatter diffraction (EBSD). In addition, we also acquired in situ oxygen isotope ratios of some mineral phases by secondary ion mass spectrometry (nanoSIMS) and Pb isotope ratios by multi-collector inductively-coupled plasma mass spectrometry (MC-ICP-MS). Our studies document, first, that Kakowa (like many L6 meteorites) contains HP phases, concentrated in and adjacent to melt veins, that require a strong shock to form. Second, we document pockets containing a series of novel minerals, including hydrous phases, that appear to be exogenous to the L6 host rock and were probably emplaced in fractures during a subsequent low-velocity collision. We use the term “exogenous” to indicate material that appears to have been added to the rock late in its history.

We argue that the low-velocity collision must have occurred after the high-velocity collision (though we have no constraint on the interval between the two events), as the hydrous phases in the exogenous material would not have survived a strong shock event. The assemblage of HP minerals and the sizes of their host MVs yield constraints on the pressure–temperature–time conditions of the strong shock experienced by Kakowa and contribute to the shock record of the L chondrites in general. It is likely, based on literature data from L chondrites, that the strong shock recorded by Kakowa was due to the large collision event that disrupted the L chondrite parent body at ~ 470 Ma^24,25^, in which case the low-velocity impact would represent the continuing collisional evolution of the resulting asteroid family after this time. Moreover, the hydrous phases in the exogenous material indicate that altered material was present in the OC-hosting region of the solar system at this late stage.

## Results

### Petrography: groundmass-melt veins—fracture

**Figure 2:**
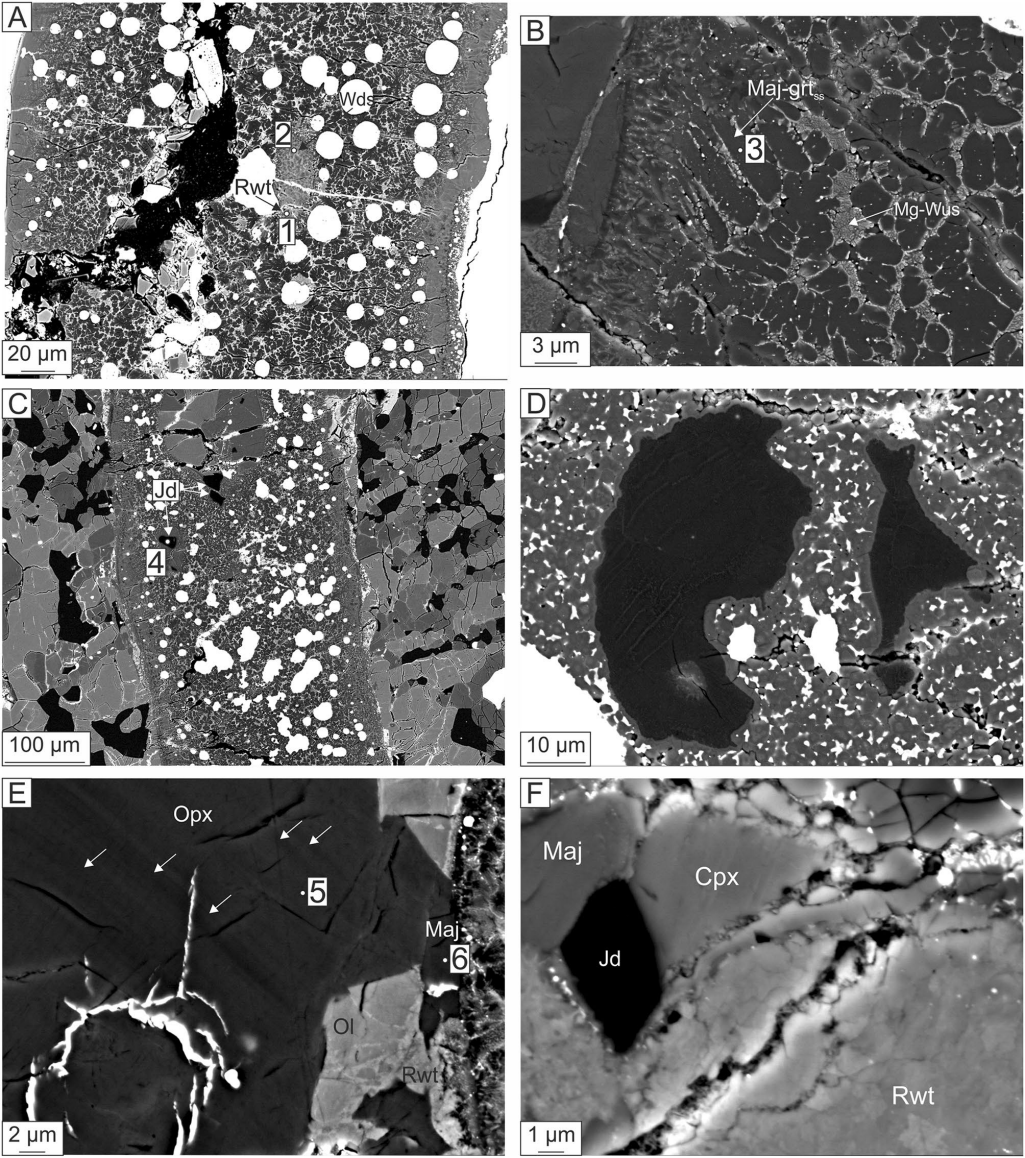
BSE images of MVs in Kakowa. (**A**) MV1a, showing ringwoodite (Rwt) (#1; Raman spectrum MV1a-1 in Fig. 5) in close association with wadsleyite (#2; Raman spectrum MV1a-2 in Fig. 5). (**B**) MV1b, with fine intergrowth of majorite-pyrope solid solution (Maj-grt) (#3, Raman spectrum MV1b-3 in Fig. 5) and magnesiowüstite (Mg-Wus). (**C**) MV2, hosting albitic jadeite (Jd) (#4; Raman spectrum MV2–4 in Fig. 5). (**D**) Glass of feldspathic composition in MV2 showing albitic jadeite lamellae. (**E**) Groundmass orthopyroxene (Opx) (#5; Raman spectrum MV1b-5 in Fig. 5) in contact with MV1b. The bands across the Opx are likely mechanical twin planes (indicated by white arrows) due to shock^5^. Further, at the contact with MV, Opx is transformed to majorite (#6; Raman spectrum MV1b-6 in Fig. 5) and olivine (Ol) is partly transformed to ringwoodite. (**F**) MV2, Albitic jadeite in contact with majorite and ringwoodite.

**Figure 3:**
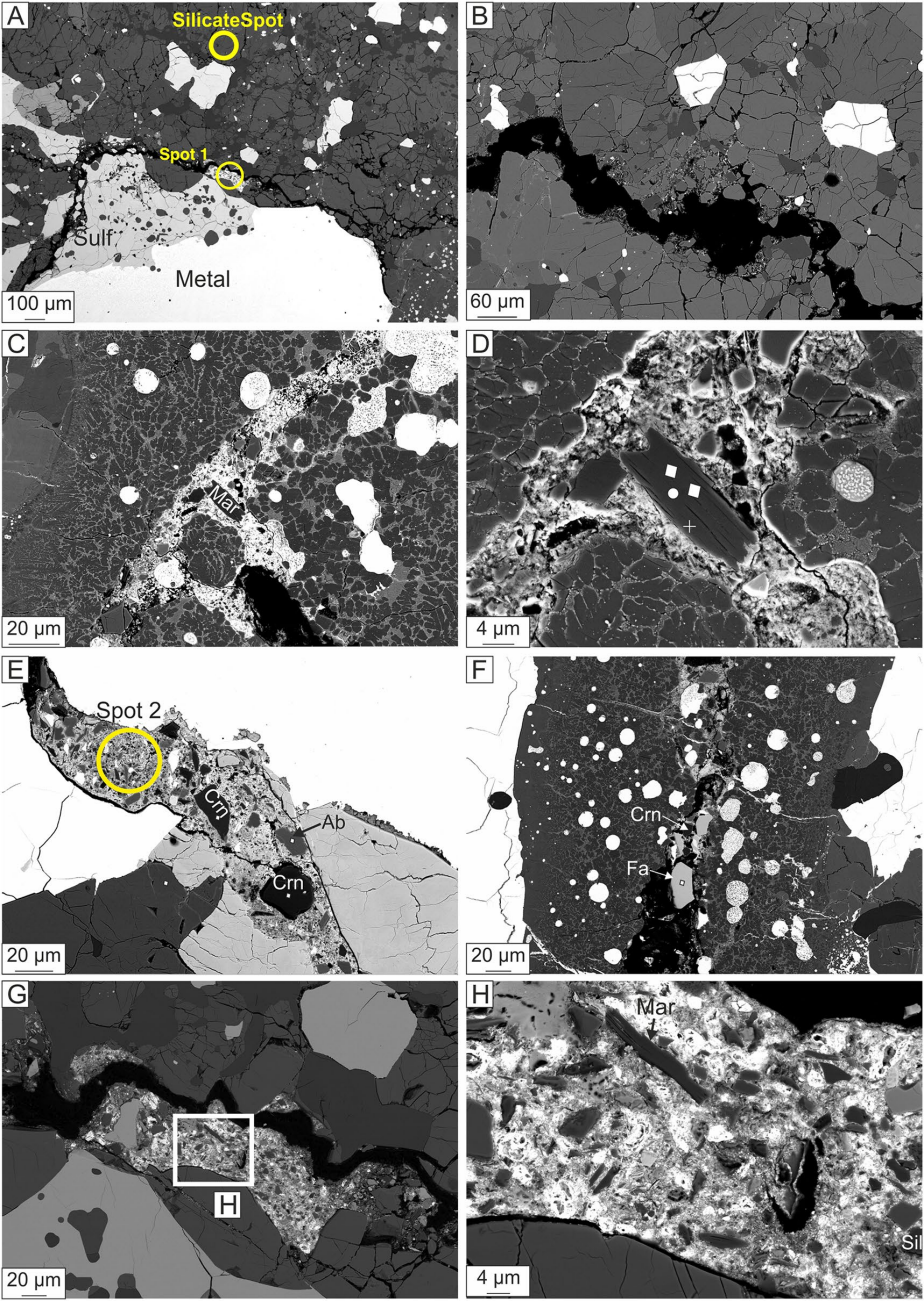
BSE images of the occurrence of exogenous material in Kakowa. (**A**) Overview showing fracture along the edge of a metal-sulfide clast, filled in part with exogenous brecciated material. “Spot 1” and “silicate spot” are areas drilled for Pb-isotope analysis. (**B**) Fracture crossing the groundmass inducing brecciation of the host minerals. (**C**) A fracture that cross-cuts the majorite-rich region of MV1b contains exogenous material including tabular crystals of margarite (Mar). (**D**) Enlargement of the margarite crystal; the white squares indicate the location of nano-SIMS O-isotope analysis points. The white circle indicates the location of margarite Raman spectrum given in Fig. 6A. The white cross indicates the location of margarite EBSD analysis given in Fig. 6C. (**E**) Corundum (Crn) and albite (Ab) in the exogenous material form subhedral to anhedral angular crystals. Spot 2 is a second region of Pb-Fe-rich matrix drilled for Pb isotope analysis. (**F**) Fayalite (Fa) in the exogenous material is also angular. (**G**) Enlargement of the patch of exogenous fracture fill shown in panel (**A**). (**H**) Further enlargement of the same patch showing margarite and a silica (Sil) phase (lower right of the figure) as well as the general texture of angular crystalline grains consolidated in a backscatter-bright matrix.

**Figure 4:**
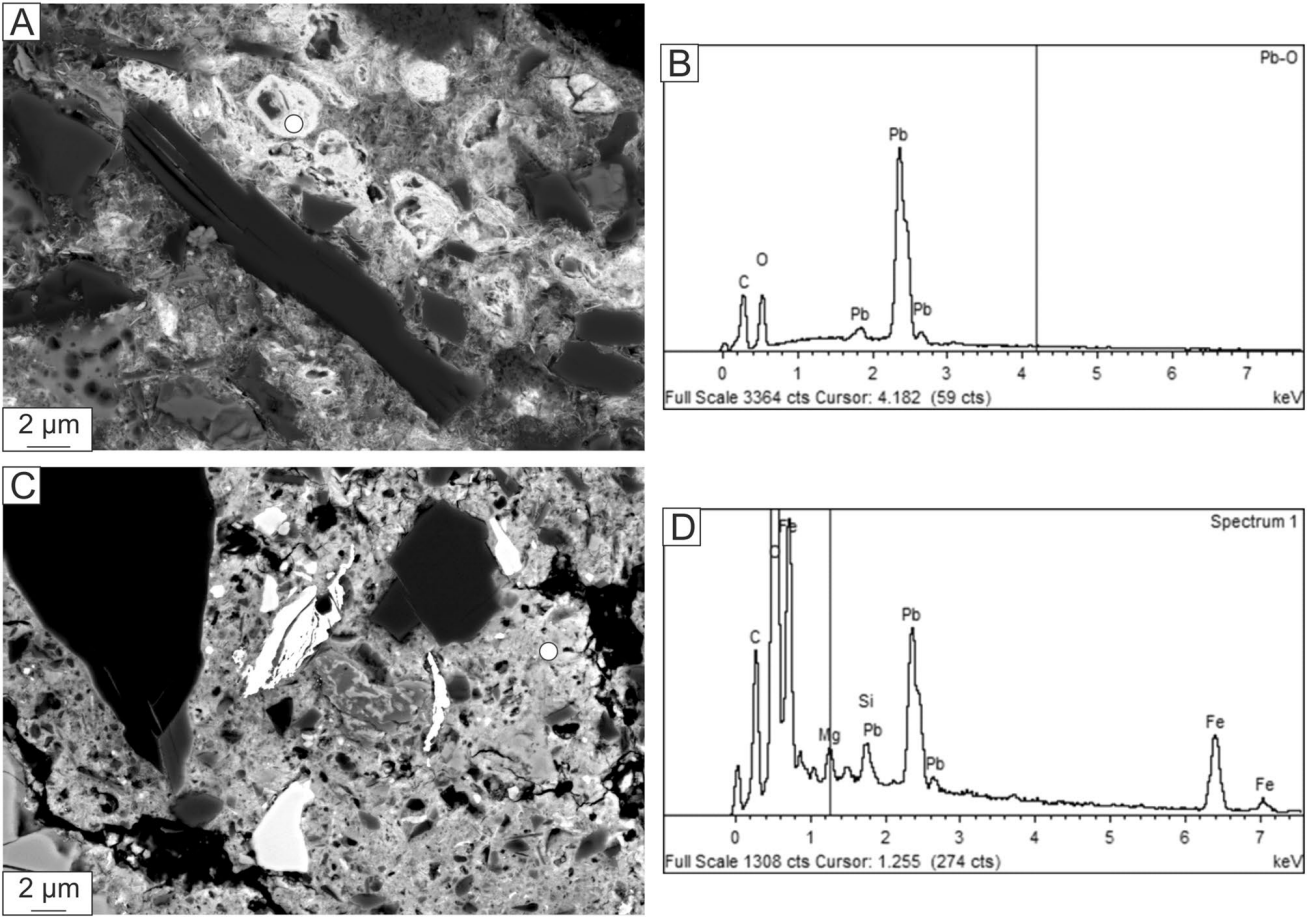
Characterization of fine-grained material from exogenous fracture fill. (**A**) BSE image and (**B**) EDS spectrum of PbO grain adjacent to margarite. (**C**) BSE image and (**D**) EDS spectrum of the Pb–Fe-rich matrix.

The gross petrography of Kakowa may be divided into chondrules, groundmass, melt veins, and fracture fill. In the groundmass, olivine grains show strong mosaicism and planar deformation features. A large fine-grained chondrule (6.1 mm in diameter) and a porphyritic chondrule (3.5 mm in diameter) dominate the studied section (NHMV-N6231); the porphyritic chondrule is bisected by the thickest MV. The three main sub-parallel MVs and minor MVs with other orientations are presumed to be the result of one shock event (Fig. 1); they are predominantly in contact with olivine but occasionally also with pyroxene and metal grains. The width of the thickest MV is nearly constant (from ~ 300 to 360 μm) across the section surface, while the thinner MVs are variable in width. The MVs consist of glass, silicate clasts (olivine, pyroxene, and plagioclase), sulfides, chromite, and Fe–Ni metal. The thick MVs are zoned from glass-bearing rims, through metal-rich crystalline layers, to silicate clast-rich cores (Figs. 2, 3). The fractures do not show any systematic structural relation with the host rock (Figs. 3, 4), however, one fracture system (about 1 cm in overall length) crosscuts the melt vein and bifurcates to surround a composite clast of Fe–Ni metal and sulfide (Fig. 3A). The fractures are mostly void space, but filling was observed in three pockets surrounding the composite clast. The fill contains a fine-grained matrix hosting angular phases (see “Fracture fill” section), likely formed as a consolidated aggregate of loose detrital grains that filled open space in the fractures (Fig. 3B).

### Melt veins

In three MV regions studied in detail, we observed the HP minerals ringwoodite, wadsleyite, majorite, and albitic jadeite (Fig. 2). Although the presence of MVs is obvious upon casual examination of the Kakowa specimen, this is the first report of HP minerals in this meteorite.

#### MV1a

The core of this melt vein is mostly a crystallized assemblage of majorite + ringwoodite + magnesiowüstite (Fig. 2A; magnesiowüstite was identified by EBSD). Aggregates of fine-grained polycrystalline ringwoodite plus wadsleyite occur locally as clasts in the MV. Along the margins of the MV, host-rock olivine is converted to polycrystalline ringwoodite, followed outwards by olivine containing ringwoodite lamellae and then by untransformed olivine. The ringwoodite zone in some places extends more than 25 μm into the host rock.

#### MV1b

A Raman spectrum obtained from a ~ 2–7 μm long grain in the core of this MV (Fig. 2B) displays the characteristic major peak at ~ 927 cm^−1^ reported from both synthetic and natural majorite^15,28,29^. The EBSD pattern collected from the same point reveals the garnet structure. EPMA analysis shows two populations of compositions among the grains with these Raman and EBSD characteristics: (a) calc-aluminous majorite with up to 4.7 wt% Al_2_O_3_, CaO in the range 1.6–2.4 wt%, and formula Na_0.05–0.09_Ca_0.12–0.19_Mg_3.22–3.35_Fe_0.45–0.67_Al_0.21–0.38_Si_3.69–3.75_O_12_; and (b) nearly end-member Fe–Mg majorite with formula Ca_0.04–0.05_Mg_3.20–3.29_Fe_0.75–0.89_Mn_0.02–0.03_Al_0.01–0.02_Si_3.87–3.92_O_12_.

#### MV2

**Figure 5:**
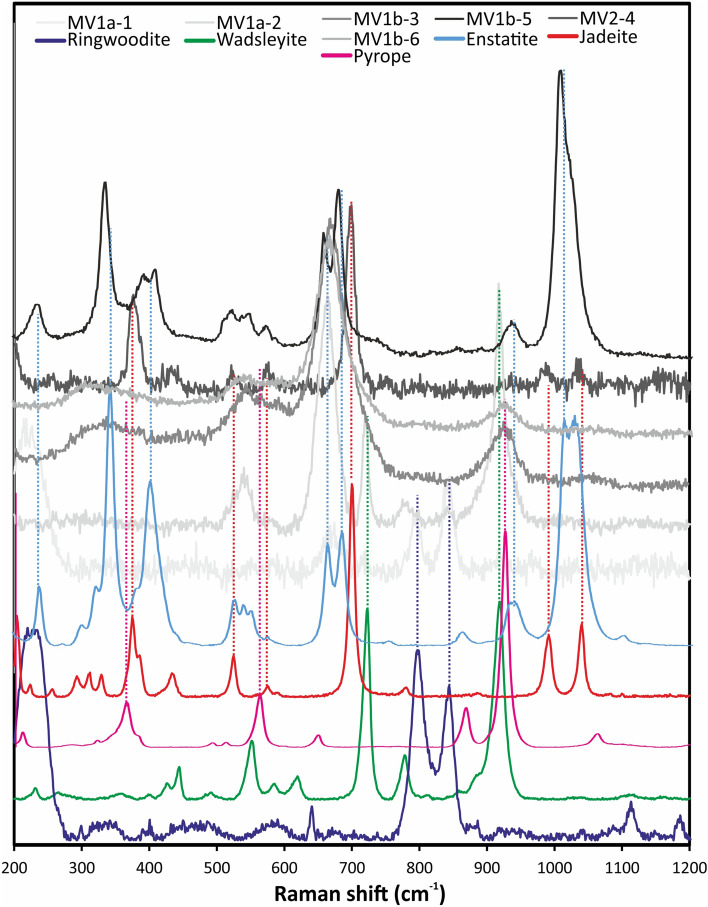
Selected Raman spectra of HP minerals in Kakowa compared to reference spectra for ringwoodite (RRUFF R070079), wadsleyite (RRUFF R090004), jadeite (RRUFF R050220.2), pyrope (RRUFF R080060), and enstatite (RRUFF R040094-3).

Irregularly shaped felsic domains in this area, up to ~ 20 μm long (Fig. 2C), mostly consist of feldspathic glass but commonly contain sub-μm parallel lamellae of a crystalline phase (Fig. 2D). The EPMA analysis of the lamellae yields the formula (Na_0.65_Ca_0.08_K_0.05_□_0.22_)(Al_0.81_Si_0.17_Fe_0.02_)Si_2_O_6_, with Ca# [100 × Ca/(Ca + Na)] of 10.5. With 22% vacant M2 sites and 17% Si on M1, this is albitic jadeite, which is beam-sensitive, like in most other published cases^30,31^. The Raman spectrum of Kakowa albitic jadeite is characteristic of clinopyroxene structure, with a distinct major peak at 698 cm^−1^ and minor peaks at 201, 376, 387, 432, 521, 574, 988, and 1035 cm^−1^ (Fig. 5). The two peaks near 1000 cm^−1^, related to vibration of [Si_2_O_6_]^4−^ groups, are resolved but not as distinct or well-separated as in the ideal jadeite spectrum. The Raman spectrum of near-endmember jadeite has major peaks at 700, 991, and 1040 cm^−1^ and minor peaks at 204, 375, 385, 433, 525, and 575 cm^−1^ (RRUFF R050220.2), which is an exceptionally good match to Kakowa even though our EPMA analysis plainly shows that the Kakowa material has albitic composition. No EBSD pattern could be obtained from this beam-sensitive material.

### Fracture fill

**Figure 6:**
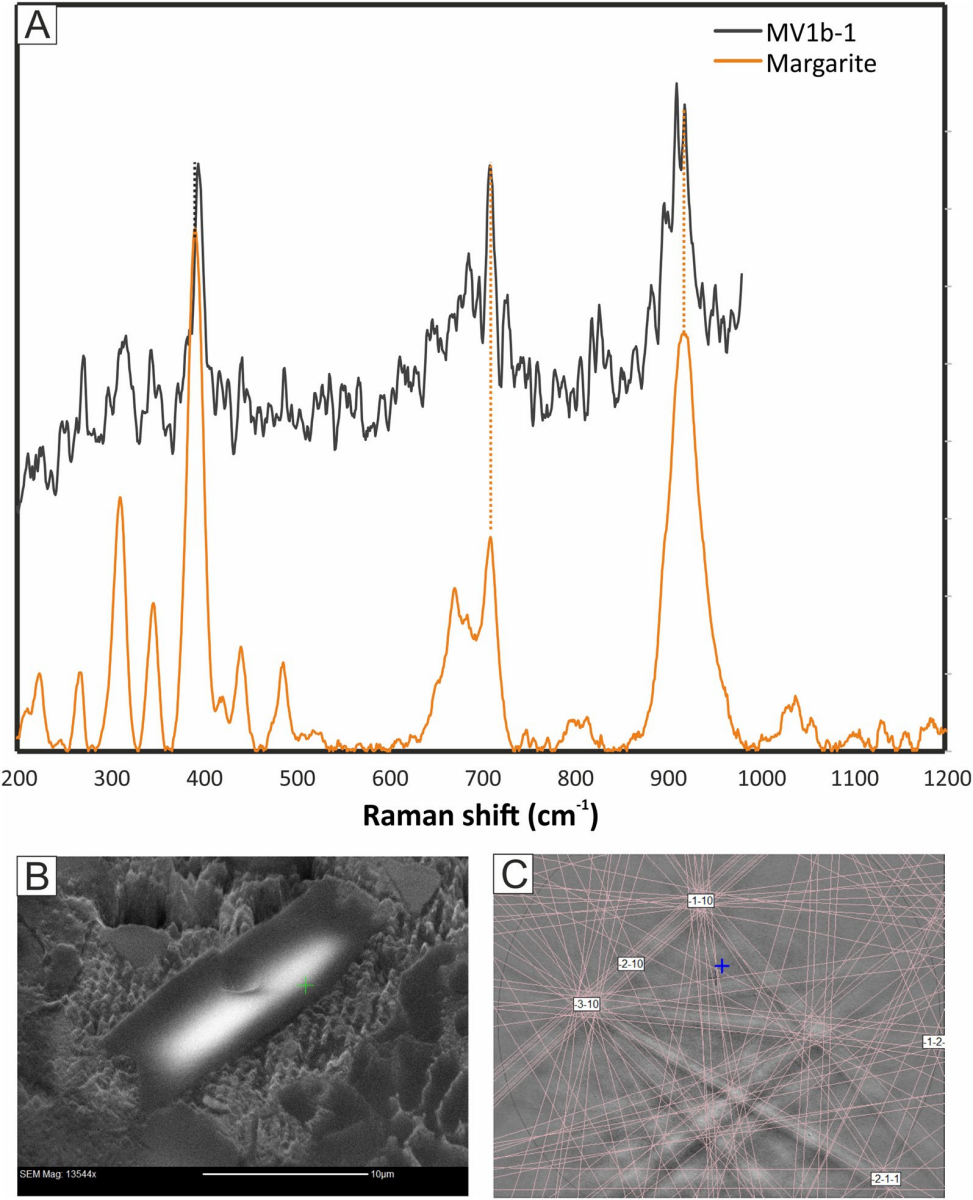
(**A**) Selected Raman spectrum obtained from exogenous margarite in the fracture fill crosscutting MV1b, compared to reference spectrum for margarite (RRUFF R060839). (**B**) Un-coated BSE image of the margarite crystal shown in Fig. 3D, during Electron back-scatter diffraction (EBSD) analysis. (**C**) EBSD pattern indexed with margarite structure.

The fractures are filled, in three pockets that we have identified, with exogenous material, composed of angular grains of corundum + fayalite + forsterite + albite + margarite + silica + FeS (troilite) + Fe–Ni-metal embedded in an Fe- and Pb-rich matrix (Figs. 3, 4). A series of energy-dispersive X-ray (EDS) analyses of the fracture-fill matrix show it to be heterogeneous in composition. Idiomorphic to subidiomorphic, 2 × 3 μm, bright crystals making up ~ 20 vol% of the fracture fill in some places are recognized as PbO. The adjacent matrix contains more than 70 wt% FeO and up to ~ 5 wt% MgO. Corundum, albite, fayalite (Fa_99–100_), and forsterite (Fa_25–26_) each occur as 10–20 μm anhedral and subhedral grains, many of them angular in shape. Margarite occurs as prismatic crystals, up to ~ 20 μm long, with a composition by EPMA that is very close to ideal: Ca_0.97_Na_0.03_Fe_0.06_Al_3.94_Si_2.02_O_10_(OH)_2_ (the hydroxyl is inferred here). The Raman spectra of these margarite grains show distinct peaks at 395, 710, 898, 911, and 919 cm^−1^ (Fig. 6A), matching very well the major peaks of the margarite reference spectrum at 392, 710, and 918 cm^−1^ (RRUFF R060839). The identification of margarite is further confirmed by EBSD (Fig. 6B,C).

### Pb isotope analysis

**Figure 7:**
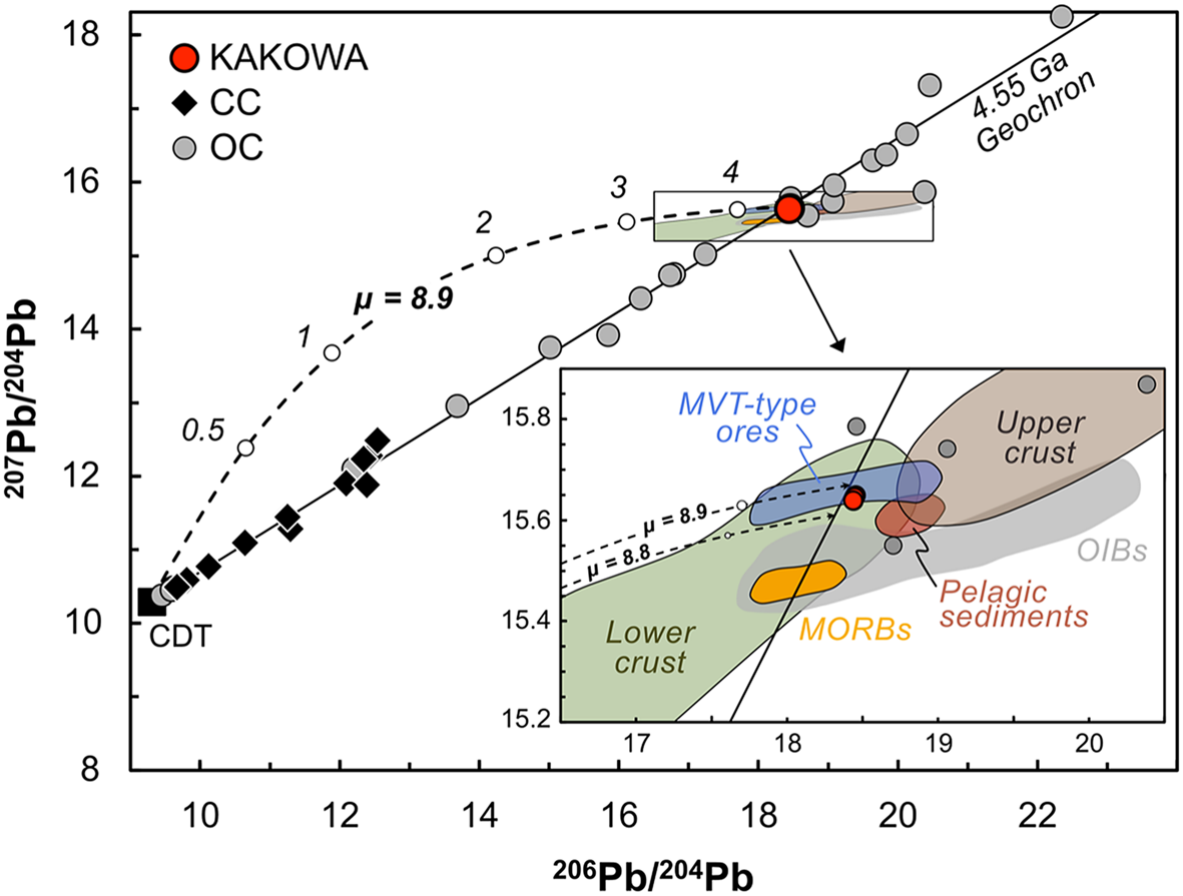
^206^Pb/^204^Pb versus ^207^Pb/^204^Pb data, plotted together with the geochron (4.55 Gyr), evolution curves for Pb-rich matrix of Kakowa, and plausible meteoritic and terrestrial Pb reservoirs. The box shows the area enlarged in the inset, where material plots if it has evolved over solar system history with μ = ^238^U/^206^Pb ~ 8.9. Inset: a close-up view showing the ^206^Pb/^204^Pb versus ^207^Pb/^204^Pb data fields for Kakowa and selected terrestrial Pb reservoirs—mid-ocean ridge basalts (MORBs), ocean island basalts (OIBs), upper continental crust, lower continental crust, pelagic sediments^48^, and MVT-type Pb ore deposits^49–52^—as well as a handful of OC meteorites that plot in this region—Kunashak, Richardton and Forest City. Meteorite data sources: Canyon Diablo troilite (CDT^53^), ordinary and carbonaceous chondrites^32,34,35,53^, and Kakowa (this study).

Three spots (each 50–100 μm in diameter) were targeted: first we drilled a spot in the silicate groundmass (Fig. 3A) as an assessment of background Pb content, and then two spots were drilled in the Pb-rich fracture-fill material (Fig. 3A,E). We obtained two orders of magnitude more Pb from drilling the exogenous fracture fill material than from the silicate matrix (Table S1). The Pb isotope ratios of the two spots in fracture fill are the same within error (Table S2): ^206^Pb/^204^Pb = 18.385, ^207^Pb/^204^Pb = 15.615, ^208^Pb/^204^Pb = 38.692 (Fig. 7). This Pb isotope composition is consistent with either ordinary chondrite (e.g., Richardton (H5) and Kunashak (L6)^32^) or terrestrial material (e.g., pelagic clay^33^) but not with carbonaceous chondrites^34,35^. Hence the Pb isotope data do not help to resolve whether the Pb is a terrestrial contaminant. However, they do help to reject the hypothesis that the fluid alteration responsible for the margarite happened on a carbonaceous body. Moreover, the data indicate a Pb isotope evolution for most of solar system history with a μ = ^238^U/^206^Pb ~ 9. Given the extreme Pb concentration of the sampled material, the data show that the U/Pb fractionation involved in making the fracture fill did not happen in early solar system history; it is consistent with an age of 470 Ma or less.

### Oxygen isotopic analysis

**Figure 8:**
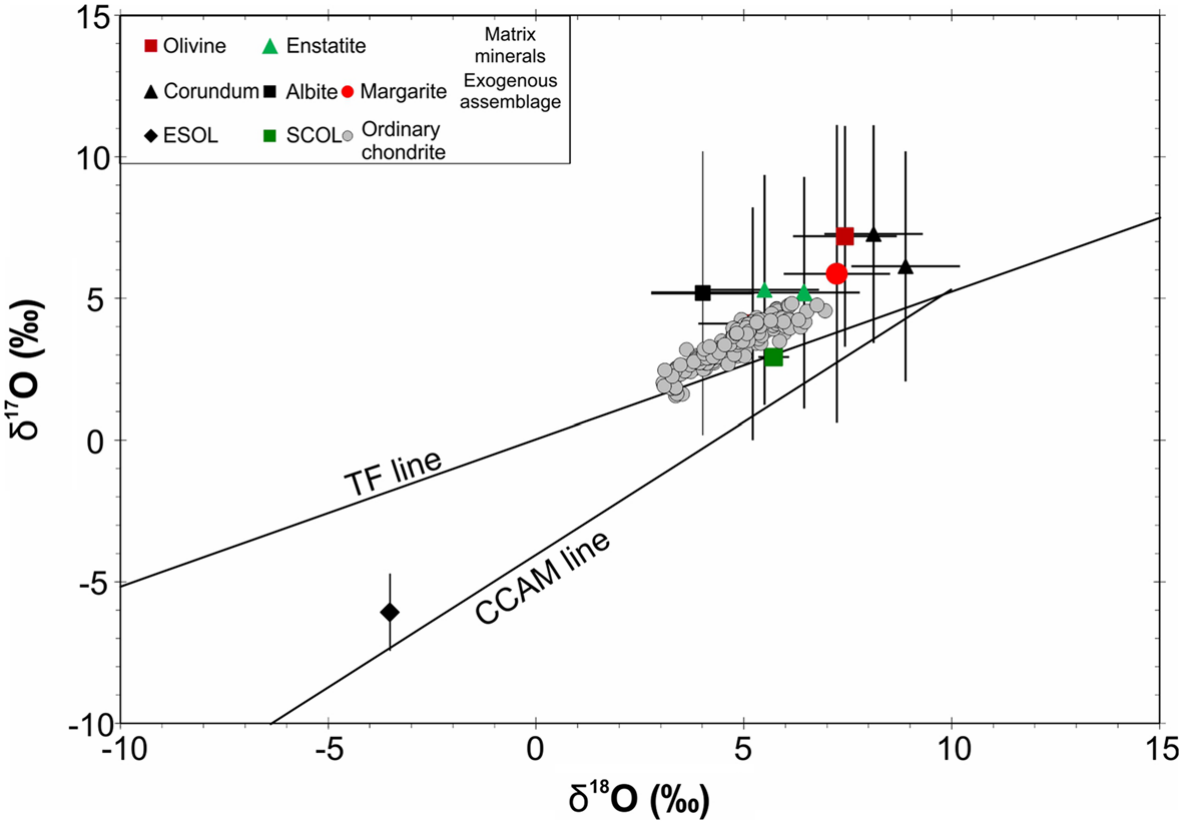
Triple oxygen isotope ratio diagram with the reference lines CCAM (Carbonaceous Chondrite Anhydrous Minerals, slope 1) and TF (Terrestrial Fractionation, slope 0.5)^54,55^, data for terrestrial and meteoritic standards (*SCOL* San Carlos Olivine, terrestrial, *ESOL* Eagle Station Olivine, a pallasite meteorite), and data from the Kakowa groundmass (olivine and enstatite) and the Kakowa exogenous fracture fill (corundum, albite, margarite). Data for ordinary chondrites are also plotted^56^. Error bars are 2σ. Δ^17^O is the vertical distance of a point from the TF line in this plot.

In a single session on the nanoSIMS, we analyzed two standard olivines (terrestrial San Carlos olivine and pallasite Eagle Station olivine), four spots on the groundmass minerals of Kakowa (two olivine and two orthopyroxene points), and four spots on minerals in the exogenous detrital fracture fill (two spots on corundum, one on albite, and one on margarite). The standard olivines match accepted values in both δ^17^O and δ^18^O, and the groundmass analyses all plot precisely within the field defined by typical L ordinary chondrite materials. This confirms that measurements during this session on the Kakowa polished section have minimal systematic errors, though we cannot quantify possible matrix effects (because all minerals are calibrated with an olivine standard). What remains in the evaluation of the exogenous phase data is the random error. The measured O isotope ratios of the detrital phases—corundum, albite, and margarite—cluster around the same OC-like region of triple oxygen isotope space as the matrix minerals (Fig. 8). All four points plot above the terrestrial fractionation line, but the 2σ errors bars on each of the four spots overlap the terrestrial fractionation line. Hence, we cannot say with confidence that any one of these analyses, in isolation, is chondritic rather than terrestrial. However, the probability that these four spots are drawn at random from a terrestrial distribution can be assessed. A Monte Carlo calculation assuming a normal distribution for counting statistical error on ^16^O, ^17^O, and ^18^O shows that the four analyzed points plot on a mass fractionation line corresponding to Δ^17^O =  + 2.5 ± 1.1‰. That is, the null hypothesis that the data are drawn from a terrestrial population is rejected at the 2.3 sigma level. There is only a 1% probability of these data arising at random from a sample of terrestrial material. The probability that they are carbonaceous chondrite material is even lower.

## Discussion

### A first, strong collision

Like many of the L chondrites, especially the L6 meteorites, Kakowa shows clear evidence of a strong shock event (Fig. 1). It is generally classified as shock stage S4–S5 because of the presence of maskelynite, shock microstructures in olivine (weak to strong mosaicism), and obvious melt veins^36,37^ (Fig. 1B). Here we document for the first time that this particular meteorite, Kakowa, contains an assemblage of preserved high-pressure phases, found within the MVs. Their mineralogy and chemistry, alongside the physical width of their host MVs, yield definite constraints on the parameters of the strong shock experienced by this particular fragment of the L-chondrite parent body.

The occurrence of wadsleyite suggests pressure (*P*) greater than 13 GPa to at most 22 GPa, whereas ringwoodite suggests an overlapping but slightly higher *P* range of 18–23 GPa; for both phases the pressure limits on their stability fields depend on temperature (*T*) and Fe content. The measured composition of majoritic garnet is consistent with *P* in the range 17–20 GPa and *T* between 1800 and 2100 °C^38^. The coexistence of all three HP phases allows for small-scale spatial or temporal heterogeneity of the *P* field.

The Raman spectrum from a Na-Si-rich glass of feldspathic composition (Figs. 2D, 3) suggests a jadeite-like pyroxene, but EPMA analysis reveals that this material is not true, stoichiometric jadeite. With vacancies on M2 and excess Si accommodated in the M1 site, this is albitic jadeite. Presently, the implications of formation of albitic jadeite for shock *P* and *T* are uncalibrated; the well-known experimental stability field of true jadeite may not be a useful guide. However, the state of the feldspars in Kakowa still yields some constraints on the shock conditions. Setting aside the preservation question, the absence of lingunite suggests maximum *P* < 21 GPa and the absence of Ca-ferrite, Ca-perovskite, or Ca-rich garnet suggests, at least locally, *P* ≤ 15.5 GPa. The presence of jadeite-like pyroxene near the center and ringwoodite at the rim of the widest MV again suggests likely temporal *P* gradients (e.g.^39^).

Concerning the time duration of the high-pressure pulse during the strong shock event, it is conventional to assume that melt veins experience local heating above the liquidus of the host rock, followed by conductive cooling due to the lower temperature matrix along their walls. Moreover, if pressure is released before cooling below the liquidus then HP phases will not be observed. In fact, temperature must drop well below the liquidus before pressure release to ensure preservation of HP phases, which are metastable at ambient pressure. Thermal models of MV cooling for the width of the widest vein—which hosts ringwoodite, majorite, and wadsleyite—suggest conductive cooling times of 26–37 ms (for details see the “Modeling strategy” section). Preservation of ringwoodite at the center of MV1 suggests temperature dropped below 1000 °C while P remained > 18 GPa^20^. Wadsleyite can grow at linear velocities ~ 1 m/s^40^, hence the observed wadsleyite crystal sizes only require the MV to spend a few μs in the wadsleyite field before quenching. The duration of a high-pressure pulse is set approximately by the ratio of the diameter of the smaller object involved in a collision to the encounter velocity, or by the two-way shock travel time across the smaller body, whichever is shorter^9^. A duration of at least 10^−3^ s^41^, given that shocks strong enough to reach peak *P* > 18 GPa travel through rock at velocity on the order of 5 km s^−1^, suggests that the smaller object involved in this collision had a diameter of at least several meters. It is difficult to provide an upper bound on this diameter; hence this result is consistent with, but does not require, the hypothesis that the strong shock resulted from the catastrophic disruption event at 470 Ma (which probably involved km-scale objects^39^).

The presence of discrete veins indicates heterogeneity of the *T* field, likely the result of collapse of spatially variable porosity during shock compression or slip along localized shear bands. It is likely an ill-defined exercise to attempt to state a single global peak *P* or *T* condition for the meteorite, much less for the maximum conditions experienced anywhere on the parent body during the associated impact event. Nevertheless, the conditions are within the range inferred from studies of HP phases, melt veins, and textures, in other L6 chondrites^16,18,20,39,42–46^.

### Is the exogenous material in Kakowa of extraterrestrial origin?

We identified pockets of detrital crystals and Pb-Fe-rich matrix filling fractures that cross-cut the melt veins formed by the strong shock (Figs. 3, 4). In principle, this fracture fill could have several sources. It could be derived from: (1) the same parent body as Kakowa, (2) a different extraterrestrial object that collided with the parent body, or (3) from terrestrial contamination. Given its consolidated detrital texture and its extended history, various parts of the fracture fill could be from more than one of these sources. Kakowa is a historical fall that was recovered within minutes of landing^27^, but to exclude terrestrial contamination (e.g., during sample preparation), we interrogated the origin of this material by micromilling areas of the fracture-fill matrix for Pb isotope analyses and by nanoSIMS in situ triple oxygen isotopic analysis of the exogenous phases. The Pb isotope results, as discussed above, are ambiguous and serve only to exclude ancient Pb enrichment and carbonaceous chondrite sources for the fracture fill matrix. The oxygen isotope results on the detrital grains are more significant. The groundmass minerals (enstatite, forsteritic olivine) consistently lie near the OC range on the three-oxygen isotope plot, above the terrestrial fractionation line (TFL). Neither the groundmass nor the detrital minerals plot on the carbonaceous chondrite anhydrous minerals (CCAM) line. The Δ^17^O values of all the measured phases from Kakowa are indistinguishable, but as a population, they are statistically separated from Δ^17^O = 0 (TFL). Therefore, the oxygen isotope results are most consistent with the detrital minerals, like the Kakowa groundmass minerals, being from an ordinary chondrite source (Fig. 8). Although corundum could be associated with CAIs from carbonaceous chondrites, it is also present in other meteorite groups^47^ and in the present case its O-isotope signature indicates an ordinary chondrite origin. We cannot resolve whether the exogenous and native components of Kakowa are from different oxygen reservoirs, but with some confidence we can exclude the hypothesis that the detrital phases are terrestrial or carbonaceous chondrite in origin.

Perhaps the most distinctive feature of the exogenous material is the presence of the hydrous calcic mica margarite. Margarite may form by hydration of anorthite, with or without corundum^57^. In the absence of corundum, the reaction yields excess SiO_2_:

1 $${\text{2CaAl}}_{{2}} {\text{Si}}_{{2}} {\text{O}}_{{8}} + {\text{ H}}^{ + } = {\text{ CaAl}}_{{4}} {\text{Si}}_{{2}} {\text{O}}_{{10}} ( {{\text{OH}}} )_{{2}} + {\text{2SiO}}_{{{\text{2aq}}}} + {\text{Ca}}^{{{2} + }}$$

whereas in the presence of corundum, margarite can form without producing silica:

2 $${\text{CaAl}}_{{2}} {\text{Si}}_{{2}} {\text{O}}_{{8}} + {\text{ Al}}_{{2}} {\text{O}}_{{3}} + {\text{ H}}_{{2}} {\text{O }} = {\text{ CaAl}}_{{4}} {\text{Si}}_{{2}} {\text{O}}_{{{1}0}} ( {{\text{OH}}} )_{{2}}$$

In the exogenous material in Kakowa, margarite coexists with both corundum and silica (Fig. 3). Albite is present but anorthite is not. Given the detrital mode of occurrence of these phases, we do not know whether margarite formed in the presence of the phases with which it now coexists. It is very likely that a Ca-bearing feldspar was the precursor, since calcic feldspars are both found as primary phases (Semarkona LL3.00^58^) and in equilibrated ordinary chondrites. Studies show that anorthite is present at degrees of thermal metamorphism up to L4 but only albite is found at L5 or higher^59,60^. Finding margarite in an L6 is therefore puzzling, except that we find it in an exogenous detrital fracture fill. The simplest explanation is that the fracture fill is derived from ordinary chondrite material that experienced thermal metamorphism of stage 4 or lower as well as fluid alteration. Moreover, the EBSD analyses of the exogenous material indicate well-crystallized minerals. This indicates that margarite is not simply a product of low-temperature aqueous alteration, which would be expected to yield poorly crystallized and fine-grained phases. Rather, the margarite indicates a two-stage process of low-temperature hydration followed by thermal metamorphism and recrystallization. Such thermal processing may have destroyed other phases that would be expected to develop during the aqueous alteration process (or these phases may remain but be too small or poorly crystalline to characterize). Yet the olivine in the exogenous material is heterogenous (forsterite and fayalite are present) and so is probably from a type 3 object that did not experience such thermal metamorphism. Hence the exogenous material itself is a detrital juxtaposition of ordinary chondrite-derived material with distinct histories, and not an equilibrated assemblage. The low-speed impactor may itself have therefore been a polymict breccia.

In principle, the fluid alteration that formed the margarite might have occurred either before or after injection of the exogenous detritus into fractures in Kakowa. However, there is no evidence (at the scale of one section) for fluid infiltration into the Kakowa groundmass, chondrules, or melt veins. There is sufficient porosity that fluids percolating through the exogenous material would likely have altered other parts of the sample as well, if alteration followed injection. Hence, we prefer a scenario in which fluid alteration and subsequent thermal maturation formed the margarite before its injection into fractures in Kakowa.

The fayalite and silica in the fracture fill are consistent with the sequence of events that we infer from the large crystalline margarite grains. Silica in achondrites has been inferred to be deposited from water during fluid alteration^61^. Thermodynamic calculations then show that the assemblage of fayalite and silica in ordinary chondrites reflects an initial event of low-temperature fluid alteration followed by thermal maturation^62^.

### Pb enrichment in fracture fill

We do not, at this time, understand the mechanism of Pb enrichment responsible for forming the PbO crystals and Pb-rich matrix of the fracture fill. Here we consider the plausible options and constraints provided by our data. The first logical explanation for the source of lead is terrestrial contamination, either before collection, during museum storage, or during preparation of the section. Our Pb isotope data do not exclude a common terrestrial source for the Pb. However, we judge that the addition of enough Pb to constitute several weight percent of the fracture fill during a few minutes^27^ between fall and collection to be highly implausible. Following King et al.^63^, a century of museum storage may lead to oxidation of minerals that are unstable in the oxidizing and water-bearing terrestrial atmosphere, such as FeS, Fe–Ni-metal, or Pb sulfates. If Pb were already present in the assemblage, it could have formed Pb oxide during storage, but the source of Pb would still likely have been extraterrestrial. The only scenario we envision in which the Pb would be entirely terrestrial in origin would be Pb metal derived from a polishing plate, later oxidized to PbO during storage of the prepared section.

The second source for the Pb that is consistent with the Pb isotope results is an ordinary chondrite reservoir. In the rare example of PbO grains at the rim of a chondrule in Chainpur (LL3.4)^64^, U–Pb systematics suggest that heating during the L-chondrite breakup event liberated Pb from Pb-bearing troilite or metal. In Kakowa, the troilite and metal in the fracture fill contain no observable Pb, which makes it difficult to judge whether all the Pb was liberated from these phases or was never present.

In any case, the problem of the Pb source, while unsolved at this time, can be considered separately from the source of the detrital grains hosted in the Pb-rich fracture fill. We rely on the oxygen isotope results from these to show that the margarite, corundum, and albite are all non-terrestrial and likely from an ordinary chondrite reservoir that experienced fluid alteration followed by thermal maturation.

### A second weak collision brings a hydrated assemblage into Kakowa

In the studied section of Kakowa, we find shock melt veins bearing high-pressure minerals that are cross-cut by fractures filled with exogenous material. We conclude therefore that Kakowa preserves a record of at least two impact events. The low-velocity impact event must come after the high-velocity impact, but we cannot constrain the time difference between them. The two events may be unrelated or, on the other hand, it could be that the low-velocity event was a secondary impact between fragments of debris from the high-velocity impact^42,65^.

Although dating of shock events can be challenging because they may only partially reset some radiometric systems, it is widely agreed on the basis of numerous studies that many L chondrites preserve a record of a strong shock at ~ 470 Ma^24,25,66–68^, commonly associated with shock darkening, formation of melt veins, and creation of HP minerals. For example, the meteorites Peace River, Taiban, Mbale, and Sixiangkou include the above shock-related features^12,25,43–46,65,69,70^. This event is so ubiquitous among L-chondrites that it is generally presumed to represent the age of catastrophic disruption of the L-chondrite parent body^24,25,71^.

Disruption of the parent body, however, need not be the last impact event experienced by its fragments. Indeed, the Ghubara L5 chondrite, for instance, contains a cognate xenolith with a slightly younger ^40^Ar/^39^Ar age than the disruption (445 Ma). The L-chondrite parent body broke into numerous asteroid fragments known as the Gefion family^71^. In turn, one or more of these fragments may have collided with other asteroids. Polymict OC meteorites, containing lithologies of various types^72^, may rarely host xenolith clasts that experienced different shock histories and preserve different shock stages (e.g., St. Mesmin meteorite^73^). In the present case, we document a further collision between a strongly shocked L6 fragment and another object with likely ordinary chondrite affinity, lower thermal metamorphic type, and a history of fluid alteration. We cannot say, with the present data, whether this object represents: (1) another fragment of the L-chondrite parent body, excavated from shallower depth and placed onto a crossing orbit with low encounter velocity, or (2) an unrelated body. The latter impact involved brecciation in the solid state, without impact heating sufficient to decompose margarite. The exogenous minerals certainly did not experience shock conditions comparable to the nearby melt veins (*P* > 18–23 GPa and *T* > 1800–2100 °C). Although impacts among planetesimals were most common in the first 100 million years of solar system history, here we infer that this impact is likely younger than ~ 470 Ma.

The data presented here are compatible with a number of detailed histories of impact and parent body disruption. For example, we cannot rule out that Kakowa records a strong shock that failed to disrupt the parent body, followed by a weak shock that nevertheless represents the disruption event. This scenario seems unlikely, however, as the weak shock is recorded by the actual presence of exogenous material from the impactor and hence it does not represent a record of a major collision elsewhere on the body, attenuated by distance. The detailed physical process (e.g., the role of fluids) by which the fill was emplaced into the fractures is not clear, but it seems likely that the maximum depth of material injection into narrow fractures during a weak impact is quite limited. Hence, we may infer that, after thermal and (strong) shock processing (perhaps during the fragmentation of the parent body), the material that would become the Kakowa meteorite was excavated to the near-surface, where it could easily be fractured and receive material transferred from a low-velocity impactor. Based on this inference, we suggest that the strong and weak shocks recorded in Kakowa did not both occur on the intact parent body. Another possibility is that yet a third impact disrupted the parent body without significantly shocking the fragment from which Kakowa originates. The most likely scenario, in our view, remains the disruption of the L-chondrite parent body at ~ 470 Ma by a major collision between bodies with high encounter velocity, recorded by the strong shock assemblage in Kakowa, followed by a low-velocity impact (recorded by the fracture and exogenous fill material) between fragments of this event, which were placed into related orbits within an asteroid family.

## Conclusions

Kakowa hosts a mineral record unique among ordinary chondrites studied to date. One of the melt veins formed during a strong shock event is cross-cut by a fracture filled by a unique exogenous material containing the hydrous phase margarite together with corundum, fayalite, forsterite, albite, and silica embedded in an Fe- and Pb-rich matrix. The mineralogy, oxygen isotope ratios, and Pb isotope ratios of the exogenous material are most consistent with derivation from an ordinary chondrite that preserves a more intense history of fluid alteration and a lower degree of thermal metamorphism than the rest of Kakowa, suggesting that alteration of the exogenous material and metamorphism of the bulk of Kakowa both predate their juxtaposition. The injection of the exogenous phases records a second impact event, with low encounter velocity, that postdates the strong shock and likely also postdates the disruption of the L-chondrite parent body.

## Material–analytical methods–modeling strategy

### Material

A single thick polished section (500 μm in thickness) of the Kakowa meteorite (NHMV-N6231) was examined for shock indicators with a focus on its melt veins (MVs). Eleven areas located in the three sub-parallel MVs were analyzed by optical microscopy, scanning electron microscopy, and electron probe microanalysis for texture and mineral chemistry (MV1 to MV11 in Fig. 1). Two of the regions (MV1 and MV2) were further studied with co-located Raman spectroscopy in order to couple structural and compositional characterization at common spots.

### Analytical methods

We used transmitted and reflected light microscopy to characterize texture, and likely mineralogy of phases large enough to be resolved optically.

*Scanning electron microscopy* (Field-emission SEM; JEOL JSM-IT300LV at NHM Vienna and Zeiss 1550VP at Caltech) yielded images, preliminary composition by energy-dispersive X-ray spectroscopy, and structure determination by EBSD. Single crystal EBSD analyses at sub-micrometer scale were performed at 20 kV and 6 nA in focused beam mode with a 70° tilted stage on uncoated sections in “variable pressure” mode (25 Pa of N_2_ gas in the chamber to reduce specimen charging). Imaging, mapping, semi-quantitative EDS analysis, and EBSD were conducted using the SmartSEM, AZtec, and Channel 5 software packages.

*Electron probe microanalyzer* yielded quantitative major element chemistry using a JEOL JXA8530F Field Emission EPMA instruments (FE-EPMA) equipped with five wavelength-dispersive spectrometers (WDS) and one energy-dispersive spectrometer (EDS) at the NHM Vienna, Austria. All the analyses were acquired using 15 kV. For minerals, a 15 nA focused beam current, 20 s counting time on peak position, and 10 s for each background were used. For glass analyses, a slightly defocused (5 μm diameter) beam, 5 nA probe current, and counting times of 10 s on-peak and 5 s on each background position were used. Natural mineral standards used were albite (Na, Si, Al), wollastonite (Ca), olivine (Mg), almandine (Fe), spessartine (Mn), orthoclase (K), rutile (Ti), chromite (Cr), and Ni-oxide (Ni) with ZAF matrix correction.

*Raman spectroscopy* analyses were collected from the polished thin section of Kakowa using a dispersive confocal Raman microscope, Renishaw inVia Reflex at the National Hellenic Research Foundation. Analyses used a 514 nm Ar-ion laser and a 100 × objective lens and spectra were collected in the region from 200–1600 cm^−1^. We acquired the spectra very carefully focusing at the surface of the samples, with a laser power ~ 5 mW to avoid destruction of the analyzing area. Acquisition time was 30–60 s averaging 5 accumulations. Additional Raman spectra were collected with a Renishaw InVia Confocal Raman microscope at the Mineral Spectroscopy Laboratory at Caltech. The 514 nm laser was set to < 2 mW power to avoid laser damage. Each spectrum was collected for 5 s with 3000 line/mm diffraction gratings, corresponding to Raman shifts of 200–1100 cm^−1^. Gaussian–Lorentzian peak fitting (fityk version 0.9.8) was used to remove background and estimate the peak centers with a precision of ~ ± 0.2 cm^−1^. Raman spectra of high-P phases, and margarite were compared with published data from RRUFF database.

*Multicollector-inductively coupled plasma mass spectrometry—Pb isotope* analysis at the Isotoparium (Caltech). The mounted section of the NHMV-N6231was micro-drilled using a GEOMILL 326 equipped with a tungsten carbide drill bit. At each of the three drill spots, three powder aliquots were recovered, by successively drilling to 10–20 μm, 50–60 μm and 80–100 μm depth. The first powder aliquot was obtained by “dry” drilling the material, and then pipetting 4–6 μL of MQ-H_2_O onto the surface to suspend the material, recovering the drop, and transferring to an acid-cleaned PFA beaker containing 1 mL of 1 M HNO_3_ (twice-distilled from ACS reagent grade HNO_3_). The second two spots were “wet” drilled by first pipetting a 4–6 μL drop onto the surface surrounding the drill bit, then drilling to suspend the released material within the drop, and finally recovering the drop and transferring to an acid-cleaned PFA beaker containing 1 mL of 1 M HNO_3_. After the final depth was drilled, an additional drop of 4–6 μL was pipetted onto and off of the surface to recover any remaining powder. The beakers containing recovered material in 1 M HNO_3_ were then placed on a hotplate at 140 °C for several hours to digest the Pb-bearing phases.

Following digestion of the Pb host phases, a 50 μL aliquot (5% of the total digest) was taken and diluted with 0.95 mL of 0.45 M HNO_3_. The concentration of Pb was checked in these solutions on a Neptune*Plus* MC-ICP-MS (Thermo Scientific) via one-point calibration with a 200 ppb Pb solution (SPEX). The samples containing > 10 ng Pb were then diluted to 15 ng/g or 6.25 ng/g for isotopic analysis. To these solutions, Tl was added to correct for instrumental mass bias^74^, such that the final solution had a 4:1 Pb:Tl ratio. Internal standard solutions were prepared at the same Pb and Tl concentrations (15 ng/g Pb + 3.75 ng/g Tl and 6.25 ng/g Pb + 1.625 ng/g Tl) using SPEX certified standards.

The Pb and Tl isotopic compositions of sample and standard solutions were analyzed on the Neptune*Plus* MC-ICP-MS using a glass spray chamber, regular sampler and skimmer cones, and a nominal 50 μL/min PFA nebulizer, yielding ~ 57 V/ppm of Pb. Each analysis consisted of 50 measurement cycles of 4.914 s in static mode, with mercury interferences monitored in cup L3 (^202^Hg), and Tl and Pb isotopes measured in L2 to H3 (L2: ^203^Tl, L1: ^204^Pb, C: ^205^Pb, H1: ^206^Pb, H2: ^207^Pb, and H3: ^208^Pb). All cups were equipped with using 10^11^ Ω amplifiers. Raw data were corrected for instrumental mass bias via external normalization with Tl^64^. For each analysis, the mass bias (β in Eq. 10 of Ref.^75^) as calculated using the measured ^203^Tl/^205^Tl ratio, a normalization ratio of 0.418922, and the respective molar masses (M203 = 202.972344 and M205 = 204.974427). The actual Pb isotope ratios were then calculated using the determined β value, the molar masses of the Pb isotopes (M204 = 203.973043, M206 = 205.964465, M207 = 206.975897, M208 = 207.976652), and the measured Pb isotope ratio (^20x^Pb/^204^Pb). Each sample solution was analyzed between five and six times. Final data are reported as the mean and 2σ of replicate analyses (between ± 0.004 and 0.012 for ^206^Pb/^204^Pb). The external reproducibility was assessed using 30 replicate analyses of SPEX Pb + Tl solutions, yielding a 2SD of ± 0.026 (2SE of ± 0.011 for n = 6) for ^206^Pb/^204^Pb.

The results (in ng Pb) obtained from the silicate spot, and the other two Pb-rich areas (Spot #1 and Spot #2), are given in Table S1. It is clearly shown that the first 10–20 μm contain little to no Pb (it seems to be removing the surface layer of glue/polish covering the sample section). The deeper drills actually sample the Pb-rich material. In the silicate spot, 2 orders of magnitude less Pb is recovered, demonstrating that the blank contamination from the drill itself is not an issue).

*NanoSIMS—oxygen isotope* analysis was carried out on a Cameca NanoSIMS 50L instrument at Caltech. A primary beam of 8 kV Cs + ions with ~ 1 pA current was used to sputter the target mineral phases in an area of 3 × 3 μm. Secondary ions of ^16^O^−^, ^17^O^−^ and ^18^O^−^ at − 8 keV were simultaneously measured with electron multipliers (EM) at mass resolving powers greater than 8000, sufficiently high to resolve the interferences with the ions of interest. A normal incidence electron gun (NEG) with accelerating potential of 8 kV was used for sample charging compensation. The data collection time of each data point was about 70 min, due to the low counting rate of ^17^O^−^. Data were corrected for the background and deadtime of the EMs. A San Carlos olivine standard was used to calibrate instrumental mass fractionation for all the mineral phases. An Eagle Station olivine standard was also used to examine the accuracy of the analysis among samples. The 2σ analytical errors for δ^17^O, δ^18^O, and Δ^17^O are ~ 3.5‰, ~ 1.5‰, and ~ 4‰, respectively.

### Modeling strategy

#### Time for complete solidification of melt veins

We approach the melt vein as a tabular shaped feature (hot slab of thickness 2*w*), surrounded by totally solid material at temperature *T*_*0*_ = 100 °C, while the interior of the vein is totally melted at temperature *T*_*m*_ = 2000 °C. The maximum time required for complete solidification of the interior melt was estimated following the procedures of Turcotte and Schubert^76^ and Langenhorst and Poirier^77^. The vein will cool and solidify in time *t*_*s*_ given by the equation

3 $$t_{{\text{s}}} = w^{{2}} /({4} \cdot \kappa \cdot \lambda^{{2}} ),$$

where κ is the thermal diffusivity and λ is a dimensionless coefficient that accounts for the boundary conditions and latent heat. The coefficient λ is obtained by numerically solving the equation

4 $$\frac{L \cdot \sqrt \pi }{{C_{p} \cdot ( {T_{m} - T_{0} } )}} = \frac{{e^{{ - \lambda^{2} }} }}{{\lambda \cdot ( {1 + {\text{erf}} [ \lambda ]} )}},$$

where *L* is the latent heat of crystallization, *C*_*p*_ is the specific heat and erf is the error function. We used for the modeling of a melt vein in Kakowa the following values: *L* = 320 kJ kg^−1^, *C*_*p*_ = 1.2 kJ K^−1^ kg^−1^, and κ = 10^−6^ m^2^ s^−1^. The temperature at the boundary with the surrounding groundmass material when the vein is solidified is given by:

5 $$T_{b} = T_{0} + \frac{{T_{m} - T_{0} }}{{1 + {\text{erf}}[ \lambda ]}}.$$

The above parameters yielded λ = 0.93, *T*_*b*_ = 1148 °C, while the cooling time was 26 and 37 ms for the minimum (300 μm) and maximum (360 μm) thickness of the thickest melt vein.

## Supplementary Information

Supplementary Tables.

## References

[1] Brearley, A. J. & Krot, A. N. Metasomatism in the early solar system: The record from chondritic meteorites. In *Metasomatism and the Chemical Transformation of Rock* 659–789 (Springer, 2013).

[2] Krot AN, Petaev MI, Nagashima K (2021). Infiltration metasomatism of the Allende coarse-grained calcium-aluminum-rich inclusions. Prog. Earth Planet Sci..

[3] Brearley AJ, Dante L, McSween HY (2006). The action of water. Meteorites and the Early Solar System II.

[4] Dobrică E, Brearley AJ (2014). Widespread hydrothermal alteration minerals in the fine-grained matrices of the Tieschitz unequilibrated ordinary chondrite. Meteorit. Planet. Sci..

[5] Lewis JA, Jones RH, Brearley AJ (2022). Plagioclase alteration and equilibration in ordinary chondrites: Metasomatism during thermal metamorphism. Geochim. Cosmochim. Acta.

[6] Stöffler D, Keil K, Scott ERD (1991). Shock metamorphism of ordinary chondrites. Geochim. Cosmochim. Acta.

[7] Stöffler D, Hamann C, Metzler K (2018). Shock metamorphism of planetary silicate rocks and sediments: Proposal for an updated classification system. Meteorit. Planet. Sci..

[8] Bischoff A, Stoeffler D (1992). Shock metamorphism as a fundamental process in the evolution of planetary bodies: Information from meteorites. Eur. J. Mineral..

[9] Beck P, Gillet P, El Goresy A, Mostefaoui S (2005). Timescales of shock processes in chondritic and martian meteorites. Nature.

[10] Gillet P, El Goresy A (2013). Shock events in the Solar System: The message from minerals in terrestrial planets and asteroids. Annu. Rev. Earth Planet. Sci..

[11] Marrocchi Y, Delbo M, Gounelle M (2021). The astrophysical context of collision processes in meteorites. Meteorit. Planet. Sci. Early View.

[12] Chen M, Sharp TG, El Goresy A, Wopenka B, Xei X (1996). The majorite-pyrope plus magnesiowustite assemblage: Constraints on the history of shock veins in chondrites. Science.

[13] Tomioka N, Fujino K (1997). Natural (Mg, Fe)SiO_3_-ilmenite and-perovskite in the Tenham meteorite. Science.

[14] Gillet P, Chen M, Dubrovinsky L, El Goresy A (2000). Natural NaAlSi_3_O_8_-hollandite in the shocked Sixiangkou meteorite. Science.

[15] Ohtani E, Kimura Y, Kimura M, Takata T, Kondo T, Kubo T (2004). Formation of high-pressure minerals in shocked L6 chondrite Yamato 791384: Constraints on shock conditions and parent body size. Earth Planet. Sci. Lett..

[16] Xie X, Minitti ME, Chen M, Mao HK, Wang D, Shu J, Fei Y (2002). Natural high-pressure polymorph of merrillite in the shock veins of the Suizhou meteorite. Geochim. Cosmochim. Acta.

[17] Xie Z, Sharp TG, De Carli PS (2006). Estimating shock pressures based on high-pressure minerals in shock-induced melt veins of L-chondrites. Meteorit. Planet. Sci..

[18] Ozawa S, Ohtani E, Miyahara M, Suzuki A, Kimura M, Ito Y (2009). Transformation textures, mechanisms of formation of high-pressure minerals in shock melt veins of L6 chondrites, and pressure-temperature conditions of the shock events. Meteorit. Planet. Sci..

[19] Baziotis IP, Liu Y, DeCarli PS, Melosh HJ, McSween HY, Bodnar RJ, Taylor LA (2013). The Tissint Martian meteorite as evidence for the largest impact excavation. Nat. Commun..

[20] Baziotis I, Asimow PD, Hu J, Ferrière L, Ma C, Cernok A, Topa D (2018). High pressure minerals in the Château-Renard (L6) ordinary chondrite: Implications for collisions on its parent body. Sci. Rep..

[21] Goodrich CA, Kring DA, Greenwood RC (2021). Xenoliths in ordinary chondrites and ureilites: Implications for early solar system dynamics. Meteorit. Planet. Sci..

[22] Day JM, Floss C, Taylor LA, Anand M, Patchen AD (2006). Evolved mare basalt magmatism, high Mg/Fe feldspathic crust, chondritic impactors, and the petrogenesis of Antarctic lunar breccia meteorites Meteorite Hills 01210 and Pecora Escarpment 02007. Geochim. Cosmochim. Acta.

[23] Hyde BC, Tait KT, Moser DE, Rumble D, Thompson MS (2020). Accretionary mixing of a eucrite impactor and the regolith of the L chondrite parent body. Meteorit. Planet. Sci..

[24] Bogard DD (1995). Impact ages of meteorites: A synthesis. Meteoritics.

[25] Korochantseva EV, Trieloff M, Lorenz CA, Buykin AI, Ivanova MA, Schwarz WH, Jessberger EK (2007). L-chondrite asteroid breakup tied to Ordovician meteorite shower by multiple isochron ^40^Ar–^39^Ar dating. Meteorit. Planet. Sci..

[26] Vokrouhlický D, Farinella P (2000). Efficient delivery of meteorites to the Earth from a wide range of asteroid parent bodies. Nature.

[27] Haidinger, W. Der Meteorit von Kakova bei Oravitza. Sitzungsberichte der Akademie der Wissenschaften mathematisch-naturwissenschaftliche Classe **34**, 11–21 (1859).

[28] Rauch M, Keppler H, Hafner W, Poe B, Wokaun A (1996). A pressure-induced phase transition in MgSiO_3_-rich garnet revealed by Raman spectroscopy. Am. Mineral..

[29] Zhang A, Hsu W, Wang R, Ding M (2006). Pyroxene polymorphs in melt veins of the heavily shocked Sixiangkou L6 chondrite. Eur. J. Mineral..

[30] Ma C, Tschauner O, Kong M, Beckett JR, Greenberg E, Prakapenka VB, Lee Y (2021). A high-pressure, clinopyroxene-structured polymorph of albite in highly shocked terrestrial and meteoritic rocks. Am. Mineral..

[31] Baziotis I, Xydous S, Papoutsa A, Hu J, Ma C, Klemme S, Berndt J, Ferrière L, Caracas R, Asimow PD (2022). Jadeite and related species in shocked meteorites: Limitations on inference of shock conditions. Am. Mineral..

[32] Huey JM, Kohman TP (1973). ^207^Pb–^206^Pb isorchron and age of chondrites. J. Geophys. Res..

[33] Patterson C (1956). Age of meteorites and the earth. Geochim. Cosmochim. Acta.

[34] Tilton GR (1973). Isotopic lead ages of chondritic meteorites. Earth Planet. Sci. Lett..

[35] Chen JH, Tilton GR (1976). Isotopic lead investigations on the Allende carbonaceous chondrite. Geochim. Cosmochim. Acta.

[36] Fritz J, Greshake A, Fernandes VA (2017). Revising the shock classification of meteorites. Meteorit. Planet. Sci..

[37] Bischoff A, Schleiting M, Patzek M (2019). Shock stage distribution of 2280 ordinary chondrites—Can bulk chondrites with a shock stage of S6 exist as individual rocks?. Meteorit. Planet. Sci..

[38] Tomioka N, Miyahara M, Ito M (2016). Discovery of natural MgSiO_3_ tetragonal garnet in a shocked chondritic meteorite. Sci. Adv..

[39] Sharp T, Xie Z, de Carli P, Hu J (2015). A large shock vein in L chondrite Roosevelt County 106: Evidence for a long-duration shock pulse on the L chondrite parent body. Meteorit. Planet. Sci..

[40] Tschauner O, Asimow PD, Kostandova N, Ahrens TJ, Ma C, Sinogeikin S, Tamura N (2009). Ultrafast growth of wadsleyite in shock-produced melts and its implications for early solar system impact processes. Proc. Natl. Acad. Sci..

[41] Miyahara M, Ohtani E, Kimura M, El Goresy A, Ozawa S, Nagase T, Hiraga K (2010). Coherent and subsequent incoherent ringwoodite growth in olivine of shocked L6 chondrites. Earth Planet. Sci. Lett..

[42] Chen M, Xie X (2008). Two distinct assemblages of high-pressure liquidus phases in shock veins of the Sixiangkou meteorite. Meteorit. Planet. Sci..

[43] Chen, M., Wopenka, B., El Goresy, A. & Sharp, T. G. High-pressure assemblages in shock melt veins in the Peace River (L6) chondrite: Compositions and pressure-temperature history. *Meteorit. Planet. Sci.* Supp. **31** (1996).

[44] Li S, Hsu W (2018). The nature of the L chondrite parent body's disruption as deduced from high-pressure phases in the Sixiangkou L6 chondrite. Meteorit. Planet. Sci..

[45] Acosta-Maeda TE, Scott ER, Sharma SK, Misra AK (2013). The pressures and temperatures of meteorite impact: Evidence from micro-Raman mapping of mineral phases in the strongly shocked Taiban ordinary chondrite. Am. Mineral..

[46] Bazhan IS, Litasov KD, Ohtani E, Ozawa S (2017). Majorite-olivine–high-Ca pyroxene assemblage in the shock-melt veins of Pervomaisky L6 chondrite. Am. Mineral. J. Earth Planet. Mater..

[47] Li JY, Zhang AC, Sakamoto N, Yurimoto H, Gu LX (2020). A new occurrence of corundum in eucrite and its significance. Am. Mineral. J. Earth Planet. Mater..

[48] Zartman RE, Doe BR (1981). Plumbotectonics—The model. Tectonophysics.

[49] Zhou JX, Wang XC, Wilde SA, Luo K, Huang ZL, Wu T, Jin ZG (2018). New insights into the metallogeny of MVT Zn-Pb deposits: A case study from the Nayongzhi in South China, using field data, fluid compositions, and in situ S-Pb isotopes. Am. Mineral. J. Earth Planet. Mater..

[50] Arribas A, Tosdal RM (1994). Isotopic composition of Pb in ore deposits of the Betic Cordillera, Spain; Origin and relationship to other European deposits. Econ. Geol..

[51] Pettke T, Oberli F, Heinrich CA (2010). The magma and metal source of giant porphyry-type ore deposits, based on lead isotope microanalysis of individual fluid inclusions. Earth Planet. Sci. Lett..

[52] Tilton, G. R., Pollak, R. J., Clark, A. H. & Robertson, R. C. Isotopic composition of Pb in central Andean ore deposits (1981). 10.1130/MEM154-p791

[53] Tatsumoto M, Knight RJ, Allegre CJ (1973). Time differences in the formation of meteorites as determined from the ratio of lead-207 to lead-206. Science.

[54] Clayton RN, Grossman L, Mayeda TK (1973). A component of primitive nuclear composition in carbonaceous chondrites. Science.

[55] Clayton RN, Onuma N, Grossman L, Mayeda TK (1977). Distribution of the presolar component in Allende and other carbonaceous chondrites. Earth Planet. Sci. Lett..

[56] Clayton RN, Mayeda TK, Goswami JN, Olsen EJ (1991). Oxygen isotope studies of ordinary chondrites. Geochim. Cosmochim. Acta.

[57] Keller LP, Buseck PR (1991). Calcic micas in the Allende meteorite: Evidence for hydration reactions in the early solar nebula. Science.

[58] Lewis JA, Jones RH (2019). Primary feldspar in the Semarkona LL 3.00 chondrite: Constraints on chondrule formation and secondary alteration. Meteorit. Planet. Sci..

[59] Kovach HA, Jones RH (2010). Feldspar in type 4–6 ordinary chondrites: Metamorphic processing on the H and LL chondrite parent bodies. Meteorit. Planet. Sci..

[60] Lewis JA, Jones RH (2016). Phosphate and feldspar mineralogy of equilibrated L chondrites: The record of metasomatism during metamorphism in ordinary chondrite parent bodies. Meteorit. Planet. Sci..

[61] Treiman AH, Lanzirotti A, Xirouchakis D (2004). Ancient water on asteroid 4 Vesta: Evidence from a quartz veinlet in the Serra de Magé eucrite meteorite. Earth Planet. Sci. Lett..

[62] Zolotov MY, Mironenko MV, Shock EL (2006). Thermodynamic constraints on fayalite formation on parent bodies of chondrites. Meteorit. Planet. Sci..

[63] King AJ, Phillips KJH, Strekopytov S, Vita-Finzi C, Russell SS (2020). Terrestrial modification of the Ivuna meteorite and a reassessment of the chemical composition of the CI type specimen. Geochim. Cosmochim. Acta.

[64] Kramer JLAM, Kik AC, Vis RD (1995). A PIXE, EPMA and SIMS study of the Chainpur meteorite: small grains of lead found in a chondrule. Nucl. Instrum. Methods Phys. Res. Sect. B.

[65] Hu J, Sharp TG (2017). Back-transformation of high-pressure minerals in shocked chondrites: Low-pressure mineral evidence for strong shock. Geochim. Cosmochim. Acta.

[66] Ormö J, Sturkell E, Alwmark C, Melosh J (2014). First known terrestrial impact of a binary asteroid from a main belt breakup event. Sci. Rep..

[67] Yin QZ, Zhou Q, Li QL, Li XH, Liu Y, Tang GQ, Jenniskens P (2014). Records of the Moon-forming impact and the 470 Ma disruption of the L chondrite parent body in the asteroid belt from U-Pb apatite ages of Novato (L6). Meteorit. Planet. Sci..

[68] Lindskog A, Costa MM, Rasmussen CØ, Connelly JN, Eriksson ME (2017). Refined Ordovician timescale reveals no link between asteroid breakup and biodiversification. Nat. Commun..

[69] McConville P, Kelley S, Turner G (1988). Laser probe ^40^Ar–^39^Ar studies of the Peace River shocked L6 chondrite. Geochim. Cosmochim. Acta.

[70] Ruzicka A, Brown R, Friedrich J, Hutson M, Hugo R, Rivers M (2015). Shock-induced mobilization of metal and sulfide in planetesimals: Evidence from the Buck Mountains 005 (L6 S4) dike-bearing chondrite. Am Min..

[71] Nesvorný D, Vokrouhlický D, Morbidelli A, Bottke WF (2009). Asteroidal source of L chondrite meteorites. Icarus.

[72] Bischoff, A., Scott, E. R., Metzler, K., & Goodrich, C. A. Nature and origins of meteoritic breccias. *Meteorites and the Early Solar System II* 679–712 (2006).

[73] Dodd RT (1974). Petrology of the St. Mesmin chondrite. Contrib. Mineral. Petrol..

[74] White WM, Albarede F, Telouk P (2000). High-precision analysis of Pb isotope ratios by multi-collector ICP-MS. Chem. Geol..

[75] Albarede F, Telouk P, Blichert-Toft J, Boyet M, Agranier A, Nelson BK (2004). Precise and accurate isotopic measurements using multiple-collector ICPMS. Geochim. Cosmochim. Acta.

[76] Turcotte DL, Schubert G (2014). Geodynamics.

[77] Langenhorst F, Poirier JP (2000). Anatomy of black veins in Zagami: Clues to the formation of high-pressure phases. Earth Planet. Sci. Lett..

