# Supplementary material for: Unique evidence of fluid alteration in the Kakowa (L6) ordinary chondrite
Source: Sci Rep. 2022 Apr 12;12:5520. doi: 10.1038/s41598-022-09465-6 (PMC9005539; doi:10.1038/s41598-022-09465-6)
Supplement: Supplementary file 1 — Supplementary Tables. [file 41598_2022_9465_MOESM1_ESM.docx]

**Supplementary info**

**Unique evidence of fluid alteration in the Kakowa (L6) ordinary chondrite**

^*^I.P. Baziotis^1^, C. Ma^2^, Y. Guan^2^, L. Ferrière^3^, S. Xydous^1^, J. Hu^2^, M. Kipp^4^, F. Tissot^4^, and P. D. Asimow^2^

^1^Agricultural University of Athens, Iera Odos 75, 11755, Athens, Greece (ibaziotis@aua.gr)

^2^California Institute of Technology, Division of Geological and Planetary Sciences, Pasadena, CA 91125, USA

^3^Natural History Museum Vienna, Burgring 7, A-1010 Vienna, Austria AT

^4^The Isotoparium, Division of Geological and Planetary Sciences, California Institute of Technology, Pasadena, CA 91125, USA

*: corresponding author

| Table S1: Results of Pb measured at different depths for the three drilled spots (silicate, #1, and #2) | | | |
| --- | --- | --- | --- |
| Depth (in μm) | Silicate Spot  (in ng Pb) | Spot #1  (in ng Pb) | Spot #2  (in ng Pb) |
| 10-20 | 0.7 | 0.7 | 2.7 |
| 50-60 | 0.4 | 13.6 | 13.5 |
| 80-100 | 0.5 | 30.8 | 10.9 |

| Table S2: Results of Pb isotopes ratios at different depths for the two spots (#1, and #2) | | | | | | |
| --- | --- | --- | --- | --- | --- | --- |
| Depth (in μm) | ^206^Pb/^204^Pb | 2σ error | ^207^Pb/^204^Pb | 2σ error | ^208^Pb/^204^Pb | 2σ error |
| Spot#1  50 μm | 18.439 | 0.012 | 15.639 | 0.010 | 38.708 | 0.026 |
| Spot#1 100 μm | 18.454 | 0.004 | 15.649 | 0.004 | 38.745 | 0.009 |
| Spot#2 50 μm | 18.446 | 0.011 | 15.645 | 0.009 | 38.731 | 0.026 |
| Spot#2  100 μm | 18.438 | 0.009 | 15.639 | 0.009 | 38.712 | 0.022 |
| Average | 18.444 |  | 15.643 |  | 38.724 |  |
